# Salting the Earth: The Environmental Impact of Oil and Gas Wastewater Spills

**DOI:** 10.1289/ehp.124-A230

**Published:** 2016-12-01

**Authors:** Lindsey Konkel

**Affiliations:** Lindsey Konkel is a New Jersey–based freelance science journalist. In June 2016 she traveled to North Dakota’s Prairie Pothole Region on a fellowship from the Institute for Journalism & Natural Resources. There she visited an active brine spill cleanup site and met with tribal members of the Mandan, Hidatsa, and Arikara Nation.

For five days in July 2014, a broken pipe spilled more than 1 million gallons of wastewater produced by unconventional oil drilling^1^ into a steep ravine filled with natural springs and beaver dams on the Fort Berthold Indian Reservation.^2^ The briny spill cut a brown swath across the North Dakota landscape, soaking into the soil and killing all vegetation in its path before it seeped into Bear Den Bay on Lake Sakakawea. This reservoir on the Missouri River is where the Mandan, Hidatsa, and Arikara Nation gets its drinking water.^3^

**Figure:**
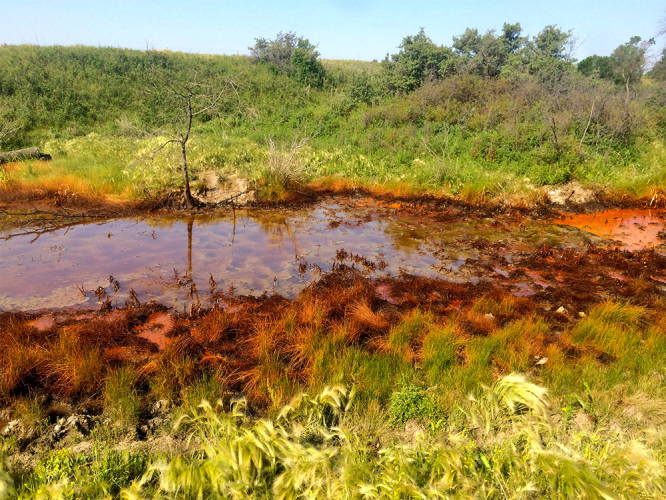
When wastewater from oil and gas extraction is accidentally or illegally released into the environment, the ecological impacts can be immediate and readily visible. Less is known about the potential human health impacts of these briny releases. © Avner Vengosh

Tribal leaders say the spill never reached the drinking water plant intake.^2^ However, for tribe members on the reservation it raised questions about the potential health impacts of leaks and spills of drilling wastewater—questions that are echoed by environmental health researchers who are calling for a closer look at the waste stream produced by oil and gas extraction.^4^

By some estimates, as much as 5% of all oil and gas wastewater produced in the United States is accidentally or illegally released into the environment.^5^ There are many potential pathways for this waste to enter surface and ground water, including spills from pipelines or tanker trucks carrying the waste, leakage from wastewater storage ponds or tanks at well pads or disposal facilities, and migration of subsurface fluids through failed well casings.^6^

Between 2009 and 2014 more than 21,000 individual spills involving over 175 million gallons of wastewater were reported in the 11 main oil- and gas-producing states of Alaska, California, Colorado, Kansas, Montana, New Mexico, North Dakota, Oklahoma, Texas, Utah, and Wyoming.^7^ In North Dakota alone, well operators have reported nearly 4,000 spills to the state since 2007.^1^

Researchers are now beginning to assess the potential impacts of these wastewater releases on the health of humans and the environment. “We know very little about the cumulative effects of these releases on the environment,” says Isabelle Cozzarelli, a hydrologist with the U.S. Geological Survey (USGS).

## The Nature of the Waste

A wealth of wastewater is produced by both conventional wells (those drilled in highly permeable rock formations) and unconventional wells (those that use hydraulic fracturing to extract oil and gas). And the dramatic increase of fracking in places like North Dakota, Ohio, and Pennsylvania in the past decade has led to a rise in the total volume of wastewater produced. In North Dakota’s Bakken shale alone, wastewater volumes more than doubled during the first few years of the fracking boom, from roughly 1.1 million gallons in 2008 to more than 2.9 million gallons in 2012.^8^

It takes a lot of water to frack a well. A single shale well may use 2–8 million gallons over its lifetime, depending on geological characteristics of the particular “play,” or formation, involved.^8^ Large volumes of fracking fluids are injected underground at high pressure. These fluids consist largely of water combined with sand or some other type of solid particle, called a proppant. The force of the injection causes fractures and fissures in low-permeability rock formations that allow trapped oil and gas to escape, and the proppant holds the fractures open.

About 1–2% of fracking fluid is a proprietary chemical mixture that performs a number of important functions in the fracking process, from increasing the viscosity of the fluid to keeping the mixture free of bacteria that could foul well passages.^9^ ^,^ ^10^ These chemicals include known or suspected endocrine disruptors, carcinogens, and other toxicants.^11^ The chemical mixture varies from well to well, and wastewater from a single well typically contains only a small fraction of the more than 1,000 known fracking chemicals.^10^ Much of the injected mixture resurfaces within the first 2 weeks after pressure is released on the well.^12^ This so-called flowback tends to look a lot like the fracking fluid mixture.

Acidizing techniques to facilitate oil and gas extraction use a similar mix of chemicals but at higher concentrations, in the range of 6–18%.^13^ While relatively uncommon, acidizing has recently gained popularity in more arid regions, such as California, where water is scarce.

Another component of the wastewater, known as produced water, occurs naturally in the rock formation and is liberated during conventional and unconventional drilling. Produced water will continue to emerge with oil and gas throughout the life of the well, and in fracked wells will pick up fracking chemicals as it flows to the surface. Produced water represents the single largest waste product associated with the oil and gas industry^5^ ^,^ ^14^—roughly 2.3 billion gallons each day.^15^

Over time, the chemistry of the produced water shifts, with no two produced waters being quite the same. Produced water may still contain small amounts of fracking chemicals.^9^ It will also typically contain a number of potentially toxic agents that occur naturally in the rock formation, which can include radioactive isotopes, organic compounds (such as benzene), ions (such as bromide, calcium, and chloride), and metals (such as cadmium, lead, and mercury).^10^ These naturally occurring constituents create concerns about safe disposal of produced water. “The [produced] water that comes up a well is potentially more harmful than the fluid used to frack it,” says Nicole Deziel, an exposure scientist at Yale University.

Sodium chloride can be a major component of produced water, which is often referred to as brine. But the salt content varies greatly from one geological formation to another and even between wells drilled in the same formation. Salinity can range from levels typical of drinking water to several times saltier than seawater.^16^

In most regions, affordable methods for treating and recycling fracking wastewater have not yet been developed,^17^ and in June 2016 the EPA finalized a rule that prevents unconventional oil and gas operators from delivering their wastewater to municipal sewage treatment plants.^18^ That is why almost all fracking wastewater is discarded offsite in injection wells.^6^ These wells are drilled into porous geologic formations such as sandstone or limestone. They may vary in depth—the defining characteristic typically is that the wells are sunk into rock formations that are isolated from drinking water sources.^19^ Nationwide there are more than 180,000 injection wells that allow oil and gas waste.^20^

However, lower-salinity water may be treated and used to irrigate farm fields or water livestock, and brines from some parts of the country can also have considerable economic value. For instance, one company with operations in Oklahoma, Texas, Kentucky, and Montana uses proprietary technology to recover iodine from fracking wastewater.^21^ However, nationwide, most drilling wastewater is discarded in deep injection wells.^22^ (Although not the subject of this story, these wells themselves may be a cause for concern—according to the USGS, deep injection of wastewater is responsible for increased earthquake activity in the central United States.^23^)

**Figure:**
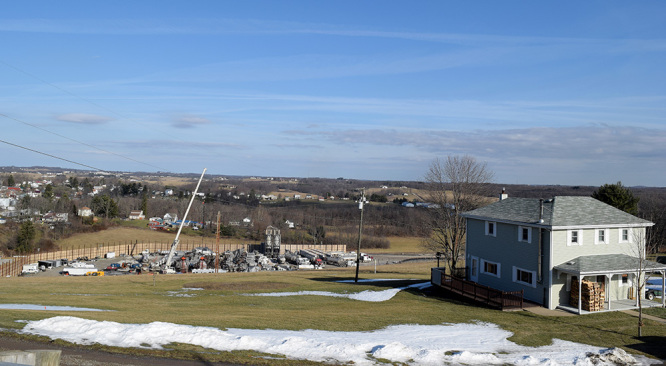
Most studies of human populations have focused on residential proximity to well pads as a proxy for exposure to drilling-associated chemicals. But studies like these can’t tell which pollutants or factors might be driving associations—or whether observed health problems are even related to oil and gas extraction. © Elise Elliott

## Assessing Exposure, Toxicity, and Risk

One of the biggest challenges in designing health risk assessments of unconventional oil and gas development may be the lack of a complete and prioritized list of chemicals on which to focus, says Deziel. “We don’t know which pollutants have the highest probability of exposure or health impact,” she says.

In 2011 the U.S. Environmental Protection Agency (EPA) began conducting research to better understand potential impacts of hydraulic fracturing on drinking water sources. A draft of the final report from the study, which was released for peer review in June 2015, included a list of 1,173 chemicals that are associated with hydraulic fracturing.^10^ The agency compiled toxicity values for cancer and noncancer effects in a publicly available draft database^24^ using governmental and intergovernmental toxicity assessments to support future risk assessments of these chemicals.

Researchers have found significant gaps in the oral toxicity data for the chemicals on the EPA list. A recent analysis showed that only 8% of the 1,076 chemicals listed as being used in fracking fluids and 62% of the 134 chemicals documented in flowback and produced water had sufficient toxicological data to calculate chronic oral toxicity values.^9^ These are values that estimate the amount of a chemical that can be ingested daily without appreciable risk of health effects.

Drilling-related wastewater spills and leaks have been shown to increase concentrations of methane and other markers of fracking-related contamination in local water supplies, as well as metals in drinking water, and salinity, radionuclides, and total dissolved solids downstream of discharges.^25^ ^,^ ^26^ ^,^ ^27^ However, these studies did not necessarily include measurements of chemicals that may be very harmful at very low concentrations, says Deziel. “We need more exposure studies measuring these types of compounds. We don’t have enough data to know whether less-toxic compounds like methane are good markers for the more complex mix of other hydraulic fracturing–related compounds,” she says.

**Figure:**
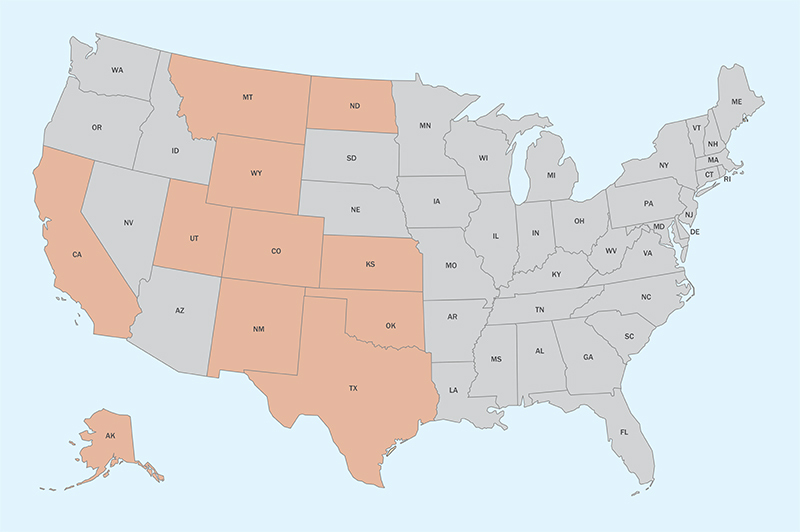
Between 2009 and 2014 more than 21,000 individual spills involving over 175 million gallons of wastewater were reported in the 11 main U.S. oil- and gas-producing states of Alaska, California, Colorado, Kansas, Montana, New Mexico, North Dakota, Oklahoma, Texas, Utah, and Wyoming (shown in orange). © Map Resources, EHP

Nevertheless, a growing body of epidemiological research has reported associations between proximity to drilling operations and adverse outcomes such as decreased semen quality and an increased risk of miscarriage, birth defects, preterm birth, low birth rate, and prostate cancer.^28^ ^,^ ^29^ ^,^ ^30^ Most studies of human populations have focused on residential proximity to drill sites as a proxy for exposure to drilling-associated chemicals. But studies like these are just a starting point, because they cannot provide insight into which pollutants or factors might be driving an association. They also cannot rule out the possibility that any given association is due to factors unrelated to oil and gas extraction.

The next step, Deziel says, is to begin collecting blood and urine from people who live near drilling operations and look to see if the chemicals measured in those biospecimens are also found in residential air and water samples. And in order to do these assessments, scientists need to know what they are looking for.

Deziel and colleagues devised a screening approach to evaluate more than 1,000 chemicals identified in fracking fluids or wastewater to prioritize those with potential human health impacts.^27^ They came up with a priority list of 67 chemicals based on known or suspected reproductive and developmental toxicity. Some of the chemicals singled out include arsenic, cadmium, lead, mercury, polycyclic aromatic hydrocarbons, benzene, toluene, and dibutyl phthalate. Having this prioritized list will help other researchers know what contaminants to look for when devising future field studies.

## Tracking Contamination in the Environment

In the Bakken Shale, most wastewater moves by pipeline to injection wells within 10 miles of a drill pad, according to industry representatives who asked not to be named. In parts of the country where drilling infrastructure coexists alongside human development, such as the Marcellus region, wastewater must often travel further for disposal.^6^ In Pennsylvania, for instance, drilling activity overwhelms local capacity for disposal in injection wells, so wastewater may be trucked to sites in Ohio or West Virginia.^6^

**Figure:**
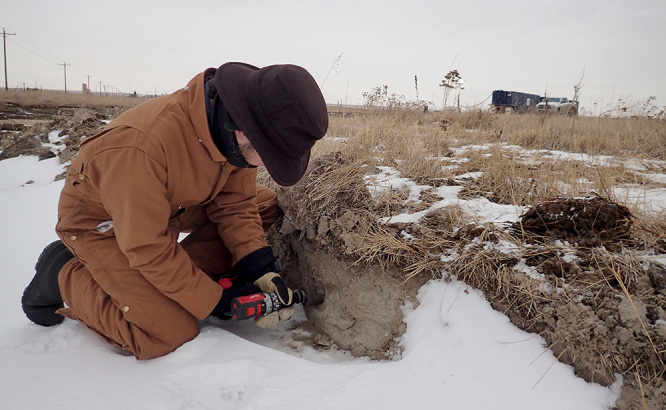
A USGS scientist collects sediment samples close to a 2015 spill at Blacktail Creek in North Dakota. Samples were collected at a depth where brine might be expected to have penetrated the soil, then were shipped to various USGS laboratories to evaluate whether the spill altered sediments and microbial communities. © Adam Benthem/USGS

**Figure:**
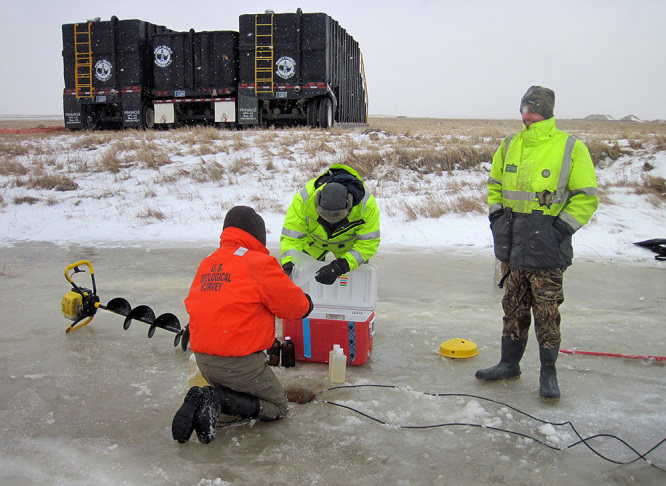
Wastewater impacts are not confined to the area immediately around a spill or leak. This USGS sampling crew has drilled through the ice to measure water quality and collect samples downstream of the Blacktail Creek spill. © Adam Benthem/USGS

Because most drilling wastewater is transported to offsite facilities, evaluating water and health impacts associated with hydraulic fracturing will mean not only looking near wells in areas with a lot of drilling, but also following the waste stream. Susan Nagel is a reproductive health scientist at the University of Missouri. In her research into the potential endocrine-disrupting effects of chemicals used in fracking, Nagel started by looking at sites where wastewater spills had occurred. “We thought those samples would be more concentrated with chemicals of interest,” she says.

Nagel and colleagues collected ground and surface water samples from sites in drilling-dense Garfield County, Colorado, where wastewater spills had occurred 2 months to 6 years earlier.^12^ Water sampled in areas with more well pads and a previous spill had higher levels of estrogenic, antiestrogenic, androgenic, or antiandrogenic activity in human cell lines than water samples taken from reference sites with limited drilling activity nearby. Yet in these sites and others, it is impossible to conclusively link the differences in water quality to oil and gas operations, because baseline environmental analyses have not been performed in most areas prior to drilling.^12^

It is possible that even in the absence of obvious spills or accidents, activities such as underground wastewater injection may have potential environmental impacts, according to Nagel. In a later study, she and colleagues including USGS geomicrobiologist Denise Akob collected surface water samples near an injection well site as well as up- and downstream from it. In assays with mammalian and yeast cells, the samples exhibited endocrine activity above levels known to cause adverse health effects for aquatic organisms. Chemical constituents of the water were consistent with wastewater from fracking operations, the researchers reported.^17^

Other studies examined the effects of exposing pregnant mice to mixtures of chemicals simulating real-world fracking wastewater. The animals drank water with 1 of 4 different concentrations of these mixtures, the 2 lowest of which were comparable to concentrations reported in drinking water near drilling sites. Reproductive effects were seen in pups at all exposures levels. Male pups showed signs of hormonal disruption including lower sperm counts, increased testis weight, and increased blood testosterone levels,^31^ while females showed reduced levels of prolactin, follicle-stimulating hormone, and luteinizing hormone.^32^

## Ecological Effects

While some researchers are studying potential human health impacts associated with unconventional oil and gas activities and produced waters, others are asking questions about ecological effects.

Akob and other USGS researchers recently quantified key biogeochemical changes associated with produced water disposal at the same West Virginia facility where Nagel’s group found signs of endocrine activity. They found that sediments collected downstream of the disposal facility were enriched with radioactive radium isotopes and contained less diverse microbial communities than upstream locations.^6^ “Microorganisms are really the base of the food chain, so they’re an important indicator of how ecology has shifted,” says Akob.

**Figure:**
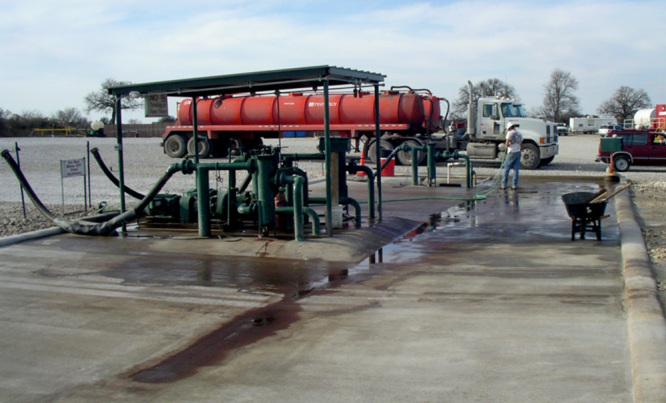
Most drilling wastewater is transported to offsite facilities such as this commercial disposal well in northern Texas. These wells are drilled into porous geologic formations that are isolated from drinking water sources. The water is stored in tanks before it is injected underground. © John Veil/Veil Environmental, LLC

**Figure:**
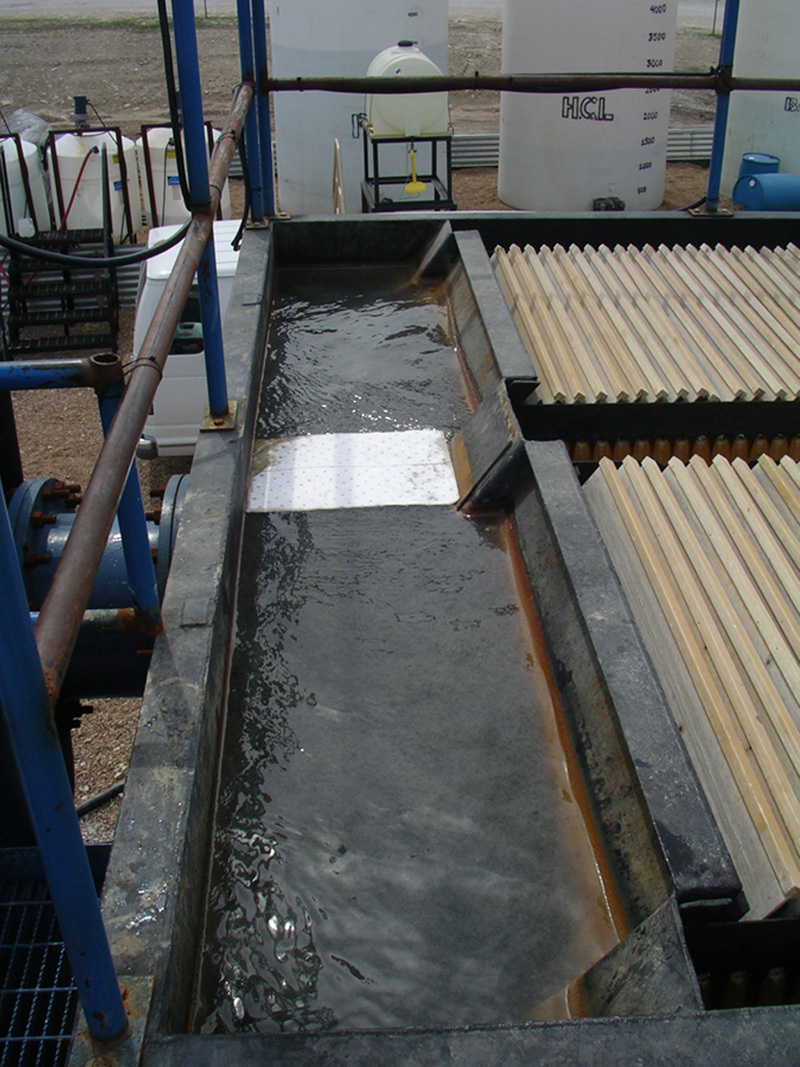
Some wastewater can be treated for reuse. At this facility in the Barnett Shale play in northern Texas, flowback water is treated and reused for new fracking fluids. An inclined plate separator removes solids from the treated flowback water. © John Veil/Veil Environmental, LLC

The study was part of a larger USGS program^33^ to characterize the broad ecological impacts of wastewater from all energy resources, not just fracking.^22^ ^,^ ^34^ “We’re trying to understand the long-term effects of these wastewater releases on the environment and develop a set of tools that others can use to analyze environmental impacts at different locations,” says Cozzarelli, the USGS hydrologist.

Most companies are very responsive in cleaning up reported spills, says Kory Richardson, refuge manager at Lostwood National Wildlife Refuge in northeastern North Dakota. Yet Richardson and other land managers are concerned about the cumulative effects on ecosystems. “No one’s out there monitoring for spills. Many of them get reported only when someone notices,” says Richardson.

Researchers at the USGS Northern Prairie Wildlife Research Center in North Dakota are trying to better understand how to track spill contamination across the state. The oil boom there overlaps an ecologically sensitive region of mixed-grass prairie and shallow wetlands called the Prairie Pothole Region.

A major concern with oil and gas development is the release of chloride-rich produced water into these wetland ecosystems.^35^ Too much salt can harm soil health. The high salinity often associated with drilling wastewater spills will kill most plant life and clog the small pores in clay soils, reducing the soil’s permeability so new plants cannot take root. “This creates a salt slick, what looks like a brown layer of asphalt across the surface,” says Kerry Sublette, a chemical engineer at the University of Tulsa.

“You can go to the North Dakota state database and see where these spills are occurring and how they are dealt with on a site-to-site basis, but we want to know about the big-picture impacts on the landscape,” says Max Post van der Burg, a USGS ecologist. Post van der Burg and colleagues tested a landscape-scale modeling approach to examine potential chloride contamination in wetlands and patterns of oil and gas development.^35^ They found that higher chloride concentrations in a wetland are associated with higher numbers of wells nearby, although, as in human studies, they can’t say with certainty that contamination from wastewater spills and leaks caused the variation in chloride levels.

## Remediating Spills

Brine spill remediation historically has focused on restoring surface soils by reversing the effects of excess salts—mainly sodium chloride. This may involve washing the salt out of the soil and countering the effects of clay dispersal with a calcium additive, or removing and replacing the contaminated soil, according to Sublette.

But excavating yards of soil can be ecologically disruptive, and rinsing the soil may force brine contamination deeper into groundwater. At one North Dakota spill site, remediation experts are piloting a new *in situ* technique to extract and remove salts from the ground without having to remove the soil itself.^36^ They are using an electrokinetic process called electromigration to separate salt molecules at the site.^37^ The process involved creating a 10-foot-deep electrical field beneath the wetland by burying 24 hexagonal bundles of electrodes to run a low-voltage current through the soil.

Chloride and sodium ions migrate toward opposite charges. “The electrical potential pulls the ions horizontally into collection wells rather than allowing them to migrate vertically into groundwater,” says Chris Athmer, an environmental scientist with Terran Corporation, the consulting firm running the project.

Athmer says the passive process, with its minimal landscape footprint, takes up to 18 months to complete, depending on a site’s size and other factors. He believes electrokinetic remediation may be a good alternative to traditional remediation techniques for environmentally sensitive areas such as wetlands or areas where it is especially important to protect a groundwater source.

It is the first time electrokinetic remediation has been used to clean up a brine spill site in the United States. The process, says Athmer, was modeled off of a similar technique called electroosmosis, which has been used successfully in Kentucky, Ohio, and Wisconsin to clean soil of industrial solvents, such as trichloroethylene.^37^

High levels of salinity associated with brine spills stress soil microbial communities, says Athmer. Changes in soil pH and temperature from the electrokinetic technique may impact soil microbes, too, although Athmer notes that changes in pH and temperature would be limited to within a few centimeters of the electrodes. He says these changes would be present only during the treatment and would likely reverse once sodium and chloride ions are removed from the soil. “Microbial communities typically thrive anywhere conditions are suitable for growth,” Athmer says. “Once near-background levels are achieved and site conditions are restored, microbial populations are expected to recover as well.”

While he cannot yet comment on the success of the pilot project, which is still ongoing, Athmer says the process is working as expected. “It is removing and is going to continue removing a lot of mass,” he says. If successful, the project could help to refine industry best practices for brine spill remediation.

“Of course the best solution is prevention,” says John Pichtel, a soil scientist at Ball State University in Indiana. “But that’s easier said than done.”
